# Concentration-Dependent Regulation of Ginger Growth and Quality by Abscisic Acid: Insights from Integrated Metabolomic and Transcriptomic Analyses

**DOI:** 10.3390/plants15081228

**Published:** 2026-04-16

**Authors:** Yifei Sun, Hui Li, Qinxi Feng, Chenrui Liu, Yunlong Li, Maoqin Xia, Chao Song, Lihui Jiang, Hong-Lei Li

**Affiliations:** 1 Chongqing Engineering Research Center for Horticultural Plant, College of Smart Agriculture, Chongqing University of Arts and Sciences, Chongqing 402160, China; 18883375351@163.com (Y.S.); lihui145327@163.com (H.L.); 13193107190@163.com (Q.F.); liucr1127@163.com (C.L.); 18983119780@163.com (Y.L.); xiamq@cqwu.edu.cn (M.X.); chaosong@cqwu.edu.cn (C.S.); 2 College of Biology and Food Engineering, Chongqing Three Gorges University, Chongqing 404020, China

**Keywords:** abscisic acid, ginger (*Zingiber officinale*), multi-omics, gingerol biosynthesis, phytohormone crosstalk

## Abstract

Abscisic acid (ABA) regulates diverse aspects of plant growth and secondary metabolism, yet its concentration-dependent effects on rhizomatous spice crops remain poorly understood at the systems level. Here, we investigated the phenotypic, physiological, hormonal, and multi-omics responses of ginger (*Zingiber officinale*) to foliar-applied ABA across a concentration gradient. Exogenous ABA modulated ginger growth in a distinctly non-linear manner. Low-to-moderate concentrations (5–15 mg/L) significantly enhanced aboveground branching and belowground rhizome yield, whereas high concentration (35 mg/L) inhibited branching while promoting structural carbohydrate accumulation, revealing a concentration-dependent trade-off between growth and secondary wall deposition. Hormone profiling uncovered global reprogramming of the endogenous hormonal network, with optimal ABA (15 mg/L) coordinately elevating growth-promoting hormones and defense-related signals, while high concentrations suppressed multiple hormone pathways and triggered negative feedback inhibition of endogenous ABA biosynthesis. Integrated metabolomic and transcriptomic analyses identified convergent enrichment on phenylpropanoid biosynthesis, gingerol biosynthesis, and plant hormone signal transduction. Co-expression network analysis revealed a highly interconnected module of 583 genes linking hormone signaling to secondary metabolism, with coordinated up-regulation of key enzymes from phenylalanine ammonia-lyase (PAL) to polyketide synthase under 15 mg/L ABA explaining the 64% increase in 6-gingerol content. This study establishes a mechanistic chain from ABA perception to improved ginger yield and quality, mediated by hormonal crosstalk and transcriptional activation of the phenylpropanoid-gingerol network. We propose an “ABA optimization window” of 5–15 mg/L for precision cultivation of high-quality ginger, providing a systems-level framework for understanding hormone-mediated regulation of secondary metabolism in medicinal and spice crops.

## 1. Introduction

Ginger (*Zingiber officinale* Roscoe), a perennial herb belonging to the genus Zingiber in the family Zingiberaceae, is one of the most widely recognized spice and medicinal plants. The species is considered to have originated in Southeast Asia and the Indian subcontinent [1,2], and is currently cultivated extensively throughout tropical and subtropical regions [3,4]. Its underground rhizome, which serves as the principal harvested organ, is valued not only for culinary use but also for its longstanding application in traditional medicine [5]. Owing to its broad pharmacological potential and economic importance, ginger has attracted increasing attention in both horticultural production and medicinal plant research [6]. The rhizome’s distinctive flavor and health-promoting properties derive from a rich array of bioactive compounds, including gingerols, shogaols, flavonoids, and essential oils [7]. These constituents confer well-documented pharmacological activities, including anticancer, antioxidant, antimicrobial, and anti-inflammatory effects [8,9,10,11], while also underpinning their growing applications in functional foods, beverages, and natural cosmetic ingredients [12,13]. As global demand for high-value, naturally sourced products continues to rise, enhancing both the yield and functional quality of ginger has become a critical imperative for the sustainable development of the industry. However, several challenges, including genetic degradation resulting from prolonged vegetative propagation, obstacles associated with continuous cropping, and the overuse of agrochemicals, threaten product safety and export competitiveness. This concern is exemplified by the detection of pesticide residues in over 66% of market samples [14]. Therefore, effective strategies to simultaneously improve both the productivity and quality of ginger are urgently needed. ABA is a sesquiterpenoid phytohormone that orchestrates diverse aspects of plant growth, development, and stress acclimation [15]. Beyond its canonical role in abiotic stress responses, ABA functions as a pleiotropic growth regulator with pronounced concentration-dependent effects [16]. While high ABA concentrations often inhibit growth, suppressing photosynthesis and reducing yield in rapeseed [17], for instance, low concentrations can promote vegetative development, nutrient assimilation, and photosynthetic efficiency [18]. This duality positions ABA as a potential tool for fine-tuning crop performance. In tuber and rhizomatous crops specifically, ABA plays pivotal roles in belowground organ development [19], assimilate partitioning, and grain-filling processes [20,21,22]. Critically, exogenous ABA application does not act in isolation; it is perceived by plants and triggers cascading changes in the endogenous hormonal network, modulating levels of auxins, cytokinins, gibberellins, and jasmonates through complex feedback and crosstalk mechanisms [23,24,25]. This hormonal reconfiguration underpins many of ABA’s pleiotropic effects on growth and metabolism.

Beyond its roles in development and hormone signaling, ABA is a potent regulator of plant secondary metabolism. Extensive research has demonstrated that exogenous ABA can enhance the accumulation of nutritionally and pharmacologically valuable compounds. In grapes, ABA treatment increases soluble sugars and anthocyanins, accelerating ripening and improving fruit quality [21]; in blueberries, it promotes anthocyanin accumulation and antioxidant capacity [25]. At the molecular level, these effects are often mediated through the transcriptional activation of key biosynthetic pathways, particularly the phenylpropanoid pathway, which serves as a metabolic hub for the production of flavonoids, lignin, and, notably in ginger, the signature pungent compounds—gingerols [7,26]. Genes encoding *PAL*, cinnamate 4-hydroxylase (*C4H*), and polyketide synthase (*PKS*) are known targets of ABA signaling in various species [27,28], suggesting a conserved mechanism for modulating phenolic metabolism.

Despite this accumulated knowledge, the application of ABA to enhance the productivity and quality of rhizomatous spice crops such as ginger remains surprisingly underexplored. To date, no systematic study has characterized the concentration-dependent effects of exogenous ABA on ginger growth, yield, and functional quality across a comprehensive dose range, nor has any research elucidated the hormonal mechanisms by which exogenous ABA reprograms the endogenous hormone network in ginger rhizomes. Furthermore, the molecular basis of ABA-mediated secondary metabolism in ginger, particularly through integrated multi-omics approaches, has yet to be investigated. These knowledge gaps represent a significant missed opportunity for developing precision cultivation strategies aimed at improving both the yield and functional quality of this economically important spice crop.

To address these gaps, we conducted a comprehensive multi-level investigation into the effects of foliar-applied ABA on ginger across a concentration gradient. We aimed to identify the optimal ABA concentration for synergistically enhancing ginger yield and functional quality, elucidate the hormonal crosstalk mechanisms through which exogenous ABA exerts its effects, and construct a systems-level model of the transcriptional and metabolic reprogramming that drives gingerol accumulation. Finally, we establish a causal chain from ABA perception to improved crop performance, thereby providing both a practical framework for precision ABA application in ginger cultivation and fundamental insights into hormone-mediated regulation of growth and secondary metabolism in medicinal plants.

## 2. Materials and Methods

### 2.1. Plant Materials and Treatments

The ginger variety used in this study was ‘Zhugenjiang’, the main cultivated variety in Southwest China. Plants were cultivated in plastic pots (diameter: 35 cm; height: 40 cm) located in a greenhouse facility at the Experimental Base of the Institute of Characteristic Plants, Chongqing University of Arts and Sciences, Yongchuan District, Chongqing, China (approximately 105.8° E, 29.3° N). The average daily temperature in the greenhouse was approximately 25 °C, while the average night temperature was maintained around 15 °C. The cultivation substrate was a mixture of nutrient soil and perlite at a volume ratio of 3:1. Each pot was filled with 4 kg of this substrate, and one piece of mother ginger (approximately 50 g) of similar size was planted per pot. When the ginger plants reached the “three-tiller” stage (approximately 80 days after planting), healthy plants with consistent growth were selected for treatment. Each treatment included six biological replicates, with one plant grown in each pot, for a total of six pots per treatment. Ginger seedlings were sprayed with ABA solutions at different concentrations (0, 5, 15, 25, and 35 mg/L, containing 0.1% (*v*/*v*) ethanol and 0.05% (*v*/*v*) Tween-20 as adjuvants). These five ABA concentrations were designated as T1 (0 mg/L, control), T2 (5 mg/L), T3 (15 mg/L), T4 (25 mg/L), and T5 (35 mg/L). Treatments commenced at the three-tiller stage and were applied once every 15 days thereafter, for a total of eight applications (at 80, 95, 110, 125, 140, 155, 170, and 185 days after planting). Spraying was carried out daily between 18:00 and 19:00 using a handheld sprayer, ensuring uniform coverage of the whole plant until leaves were moist but not dripping. Apart from the spray treatments, all other cultivation management practices were kept consistent across all groups. Plants were harvested 200 days after planting. At this stage, both aboveground and belowground agronomic traits were systematically evaluated using six biological replicates. Fresh rhizome samples were also collected in three biological replicates, immediately frozen in liquid nitrogen, and stored at −80 °C for subsequent analyses.

### 2.2. Determination of Agronomic Traits

Agronomic traits were assessed in accordance with the *Descriptors and Data Standard for Ginger* [26,27]. For phenotypic evaluation, six pots containing uniformly growing plants were randomly selected from each treatment. The measured aboveground traits included plant height, pseudostem diameter, tiller number, leaf number, leaf length, and leaf width. At harvest (200 days after planting), belowground traits were further determined, including rhizome length, rhizome width, rhizome number per plant, rhizome diameter, fresh rhizome weight, and total fresh biomass per plant. Mean values from six biological replicates were used for statistical analysis.

### 2.3. Determination Methods for Photosynthetic Parameters and Chlorophyll Content

On days 1, 3, 6, and 9 after the initial application of exogenous ABA, healthy ginger plants with uniform growth were selected from each treatment group between 9:00 AM and 11:00 AM. Photosynthetic parameters of the leaves, including net photosynthetic rate (Pn), stomatal conductance (Gs), transpiration rate (Tr), and intercellular CO_2_ concentration (Ci), were measured using a LI-6400XT portable photosynthesis system (LI-6400XT, Beijing Ecotek Technology Company Limited, Beijing, China) with three biological replicates. Concurrently, relative chlorophyll content in the leaves was measured using a SPAD meter (Zhejiang Top Instrument Co., Ltd., Hangzhou, China) with six biological replicates. The mean values were calculated for subsequent analysis.

### 2.4. Determination Method for Endogenous Hormone Content

(1) Metabolite Extraction

Approximately 100 mg of frozen rhizome tissue was accurately weighed into a 2 mL centrifuge tube. Steel balls and 1 mL of ice-cold isopropanol-water (80:20, *v*/*v*) containing an isotope-labeled internal standard mixture [28] were added. After vortexing for 30 s, the mixture was homogenized at 40 Hz for 4 min and ultrasonicated in an ice-water bath for 5 min. The homogenization and ultrasonication steps were repeated three times. Samples were then incubated at −20 °C for 1 h, followed by centrifugation at 14,000× *g* for 15 min at 4 °C. An 800 µL aliquot of the supernatant was collected and evaporated to dryness under a gentle nitrogen stream. The residue was reconstituted in 160 µL of methanol-water (50:50, *v*/*v*), vortexed thoroughly, and centrifuged at 14,000× *g* for 10 min at 4 °C. The supernatant was filtered through a 0.22 µm membrane filter prior to LC-MS analysis.

(2) Preparation of Standard Solutions

Appropriate amounts of each standard were accurately weighed and placed into volumetric flasks to prepare individual standard stock solutions at a concentration of 1000 ng/mL. Suitable aliquots of each individual stock solution were transferred into a 10 mL volumetric flask and made up to volume with solvent to prepare a mixed standard solution. This mixed standard solution was then serially diluted to prepare a series of calibration solutions with a concentration gradient. All calibration solutions were spiked with the isotope-labeled internal standard mixture at the same final concentration as in the test samples.

(3) UHPLC-MRM-MS Analysis

Chromatographic separation was performed using a Waters ACQUITY I-Class UPLC system equipped with an ACQUITY UPLC^®^ HSS T3 column (100 mm × 2.1 mm, 1.8 µm, Waters Corporation, Milford, MA, USA). The mobile phase consisted of A: water containing 0.1% formic acid, and B: acetonitrile containing 0.1% formic acid. The column temperature was maintained at 40 °C, the autosampler temperature was set to 10 °C, and the injection volume was 5 µL.

Mass spectrometric analysis was conducted using a SCIEX QTRAP^®^ 6500+ triple quadrupole mass spectrometer (SCIEX, Framingham, MA, USA) equipped with an IonDrive™ Turbo V electrospray ionization (ESI) source (SCIEX, Framingham, MA, USA). The ion source parameters were set as follows: curtain gas pressure 35 psi, ion source gas 1 pressure 50 psi, ion source gas 2 pressure 55 psi, ionization temperature 550 °C; spray voltage +5500 V in positive ion mode and −4500 V in negative ion mode.

Standard solutions for each analyte were introduced into the mass spectrometer via flow injection to optimize multiple reaction monitoring (MRM) parameters. For each target compound, several precursor-product ion pairs with the highest response signals were screened, and their declustering potential (DP) and collision energy (CE) were systematically optimized. The ion pair exhibiting the optimal response was selected for final quantitative analysis.

### 2.5. Determination Methods for Ginger Quality Indicators

Gingerol content was determined using High-Performance Liquid Chromatography (HPLC). A total of 0.5 g of powdered ginger sample (accurate to 0.0001 g) was weighed into a 5 mL centrifuge tube. Then, 2 mL of 80% aqueous methanol was added, and the sample was homogenized. The homogenate was transferred to a 15 mL centrifuge tube, and the volume was adjusted to 5 mL with 80% aqueous methanol. The mixture was extracted in a 66 °C water bath for 2 h. After cooling, the extract was centrifuged at 4000× *g* for 5 min. The extraction was repeated once, and the two supernatants were combined, mixed well, and filtered through a 0.22 µm membrane filter. A 6-gingerol standard (purity ≥ 98%, CAS: 23513-14-6, Sigma-Aldrich Trading Co., Ltd., Shanghai, China) was used to generate calibration curve. The chromatographic conditions were as follows: a UV detector (VWD, 280 nm), a Thermo C18 column (250 mm × 4.6 mm, 5 µm, Thermo Fisher Scientific lnc., Waltham, MA, USA), column temperature of 25 °C, and an isocratic mobile phase of acetonitrile:methanol:water (50:5:45, *v*/*v*/*v*) delivered at a flow rate of 1 mL min^−1^; the injection volume was 20 μL [29].

### 2.6. Plant Wide-Target Metabolomics Analysis

Samples from the five treatment groups were subjected to metabolomics and transcriptomics analysis. After vacuum freeze-drying, 50 mg of each sample was weighed and placed into a tube. Then, 1000 µL of extraction solution (methanol:acetonitrile:water, volume ratio 1:2:1) was added, and the mixture was vortexed for 30 s. Steel balls were added, and the sample was processed in a grinder at 45 Hz for 10 min, followed by ultrasonication for 10 min (in an ice-water bath) [30]. The sample was allowed to stand at −20 °C for one hour and then centrifuged at 12,000 rpm for 15 min at 4 °C. Carefully, 300 µL of the supernatant was taken and filtered through a 0.22 µm organic membrane filter into a 2 mL injection vial. A 10 µL aliquot from each sample was mixed to create a quality control (QC) sample for machine injection detection. The LC-MS system used for metabolomics analysis consisted of a Waters Acquity I-Class PLUS UPLC system (Waters Corporation, Milford, MA, USA) coupled with an AB Sciex Qtrap 6500+ high-sensitivity mass spectrometer (AB Sciex Pte. Ltd., Framingham, MA, USA). The chromatographic column used was a Waters Acquity UPLC HSS T3 column (1.8 µm, 2.1 × 100 mm, Waters Corporation, Milford, MA, USA). Mass spectrometry conditions were as follows: Electrospray Ionization (ESI) source temperature 550 °C; Ion Spray Voltage (IS) 5500 V (positive ion mode)/−4500 V (negative ion mode); Ion Source Gas I (GSI), Gas II (GSII), and Curtain Gas (CUR) were set to 50, 55, and 35 psi, respectively; the collision-induced dissociation parameter was set to Medium. Instrument tuning and mass calibration were performed using 10 and 100 µmol/L polypropylene glycol solutions in QQQ and LIT modes, respectively. QQQ scanning was conducted using Multiple Reaction Monitoring (MRM) mode with collision gas (nitrogen) set to Medium [31]. The declustering potential (DP) and collision energy (CE) for each MRM ion pair were optimized. Based on the metabolites eluting in each period, a specific set of MRM ion pairs was monitored to ensure analytical accuracy and sensitivity. Qualitative and quantitative analysis of sample metabolites was performed using the GB-PLANT database from BMK Cloud (Beijing) Biotechnology Co., Ltd., Beijing, China. Characteristic ions for each substance were selected using the triple quadrupole, and their signal intensities were acquired by the detector. After obtaining the mass spectrometry data for metabolites from different samples, peak area integration was performed for all substance mass spectral peaks, and integration correction was applied for the same metabolite peaks across different samples. The repeatability within groups and among control samples was assessed using Principal Component Analysis (PCA) and Spearman correlation analysis. Identified compounds were classified and annotated with pathway information using the KEGG (http://www.genome.jp/kegg/), HMDB (https://hmdb.ca/), and LipidMaps (https://lipidmaps.org/) databases. Based on the grouping information, the fold change (FC) for each compound was calculated, and its significance was evaluated using a T-test (*p*-value). For further analysis, Orthogonal Partial Least Squares Discriminant Analysis (OPLS-DA) modeling was performed using the ropls package in R language, and the reliability of the model was validated through 200 permutation tests. The Variable Importance in Projection (VIP) values from the model were calculated through multiple cross-validations. Differential metabolites were screened by combining FC, *p*-value, and VIP value, applying the criteria of FC > 1, *p*-value < 0.05, and VIP > 1 [32]. KEGG pathway enrichment analysis for differential metabolites was performed using hypergeometric distribution tests to assess significance.

### 2.7. Illumina Transcriptome Sequencing and Analysis

Total RNA was extracted from plant samples using the RNA prep Pure Plant Kit (Tiangen, Beijing, China) according to the manufacturer’s instructions. RNA concentration and purity were measured using a NanoDrop 2000 spectrophotometer (Thermo Fisher Scientific lnc, Wilmington, MA, USA). RNA integrity was assessed using the RNA Nano 6000 Assay Kit for the Agilent Bioanalyzer 2100 system (Agilent Technologies Inc., Santa Clara, CA, USA). For library construction, 1 µg of high-quality RNA per sample was used with the Hieff NGS Ultima Dual-mode mRNA Library Prep Kit for Illumina (Yeasen Biotechnology (Shanghai) Co., Ltd., Shanghai, China). The prepared libraries were sequenced on the Illumina NovaSeq platform (Illumina, Inc., San Diego, CA, USA), generating 150 bp paired-end reads.

Raw data in Fastq format were initially processed using in-house Perl scripts. In this step, clean data were obtained by removing reads containing adapters, reads containing poly-N regions, and low-quality reads from the raw data. Concurrently, the Q20, Q30, GC content, and sequence duplication levels were calculated. All downstream analyses were based on high-quality clean data.

The clean reads were aligned to the reference genome sequence. Based on the reference genome, only reads with perfect matches or a single mismatch were used for further analysis and annotation. The Hisat2 tool was employed for aligning reads to the reference genome. StringTie was used to identify known transcripts and predict novel transcripts from the Hisat2 alignment results, utilizing the reference annotation-based transcript (RABT) assembly method. Transcripts were annotated using the following databases: Nr (NCBI non-redundant protein sequences); Pfam (Protein family); KOG/COG (Clusters of Orthologous Groups of proteins); Swiss-Prot (A manually annotated and reviewed protein sequence database); KO (KEGG Ortholog database); GO (Gene Ontology). Differential expression analysis was performed with criteria of Fold Change ≥ 2 and False Discovery Rate (FDR) < 0.01.

### 2.8. Data Processing

All experimental data were expressed as mean ± SD. Six biological replicates per treatment were used for aboveground and belowground agronomic traits and chlorophyll content, and three biological replicates per treatment were used for all other measurements. Statistical analyses were performed using IBM SPSS Statistics 27.0.1 (IBM Corp., Armonk, NY, USA). Differences among treatments were evaluated by one-way analysis of variance (ANOVA), followed by Duncan’s multiple range test for mean separation. A value of *p* ≤ 0.05 was considered statistically significant. Figures were generated using OriginPro 2024 (OriginLab Corporation, Northampton, MA, USA) and GraphPad Prism 10.1.2 (GraphPad Software, Boston, MA, USA).

## 3. Results and Analysis

### 3.1. Effects of Exogenous ABA on Ginger Growth

#### 3.1.1. Aboveground Growth Responses to Exogenous ABA

**Figure 1:**
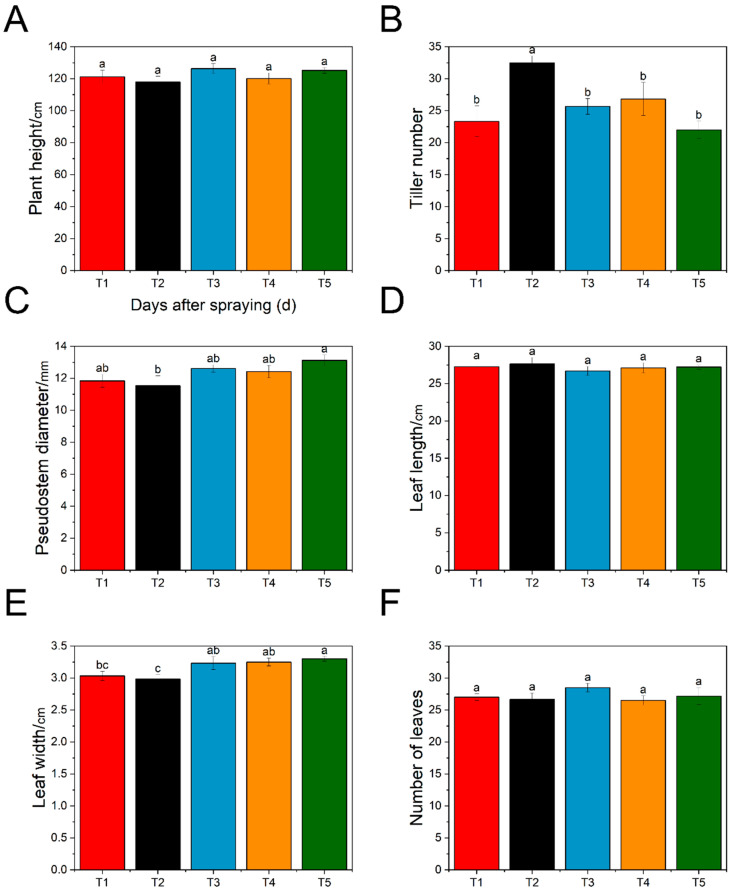
Effects of exogenous ABA on aboveground traits of ginger at final harvest (200 days after planting). (**A**) Plant height; (**B**) tiller number; (**C**) pseudostem diameter; (**D**) leaf length; (**E**) leaf width; and (**F**) number of leaves on the main stem. Data are presented as the mean ± SD of six biological replicates. Different lowercase letters indicate significant differences among treatments at *p* ≤ 0.05. T1, 0 mg/L ABA (control); T2, 5 mg/L ABA; T3, 15 mg/L ABA; T4, 25 mg/L ABA; and T5, 35 mg/L ABA.

At the final harvest (200 days after planting), exogenous ABA exerted distinct effects on the aboveground agronomic traits of ginger. No significant differences were detected among treatments (T1–T5) in plant height (Figure 1A), leaf length (Figure 1D), or leaf number (Figure 1F). In contrast, tiller number (Figure 1B) was the most responsive trait, reaching its highest level under the T2 treatment and differing significantly from the other treatments. For pseudostem diameter (Figure 1C) and leaf width (Figure 1E), the T5 treatment showed the highest values, whereas relatively lower values were observed under T2. Taken together, these results indicate that lower ABA concentrations favored tiller formation, whereas higher concentrations were more effective in promoting pseudostem thickening and leaf expansion. By comparison, plant height, leaf length, and leaf number were only marginally affected by ABA treatment. These findings suggest that exogenous ABA, particularly at 5–15 mg/L, may promote aboveground growth mainly through stimulation of tillering, which could increase the number of photosynthetically active organs and thereby enhance assimilate production capacity.

#### 3.1.2. Belowground Rhizome Development in Response to ABA

**Figure 2:**
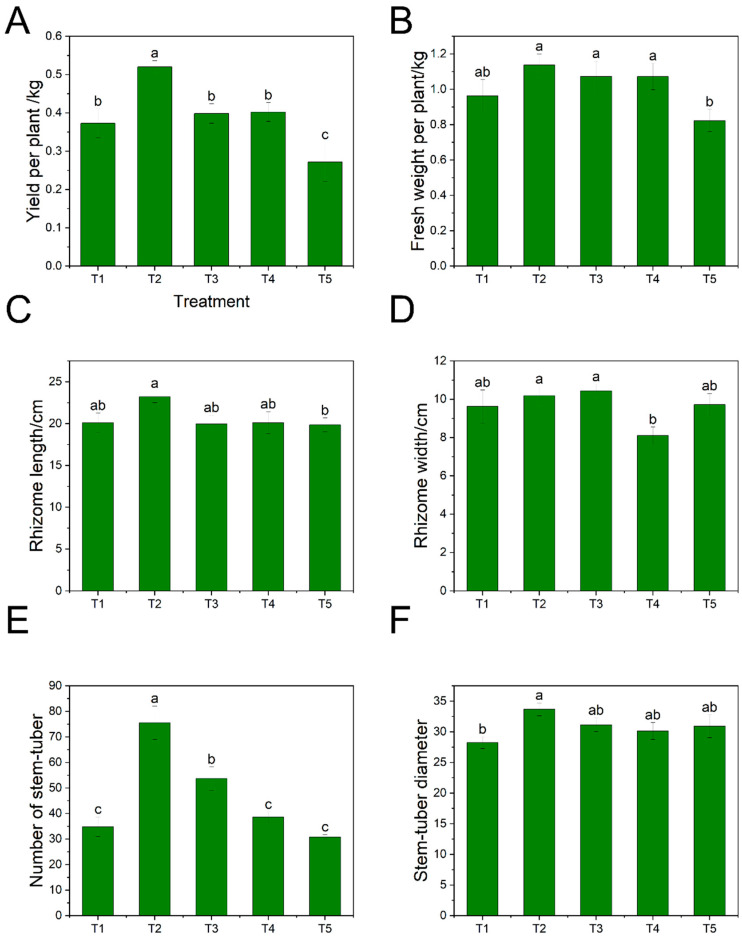
Effects of Exogenous ABA on Underground Growth Morphological Indicators of Ginger. (**A**) Yield per plant, (**B**) whole-plant fresh weight, (**C**) rhizome length, (**D**) rhizome width, (**E**) number of stem-tubers, (**F**) stem-tuber diameter. Data are presented as mean ± SD from six replicates. Different letters (a, b, c) indicate significant differences at *p* ≤ 0.05. T1: 0 mg/L ABA (control), T2: 5 mg/L, T3: 15 mg/L, T4: 25 mg/L, T5: 35 mg/L.

Yield per plant and whole-plant fresh weight were most significantly enhanced by the T2 treatment (Figure 2). This optimal concentration increased average yield per plant to 0.52 kg and stem-tuber diameter to 33.66 mm, alongside a substantial increase in the number of stem-tubers (averaging 75.5 stem-tubers), ranking T2 > T3 > T4 > T1 > T5 for both yield and stem-tuber count (Figure 2A,B,E,F). The T3 treatment also promoted stem-tuber number (averaging 53.67 stem-tubers), although this did not translate into a statistically significant yield increase, possibly due to compensatory effects on individual stem-tuber size (Figure 2A,E). In contrast, higher concentrations (T4, T5) did not significantly affect any of the measured rhizome parameters compared to the control, suggesting that the promotive effect on belowground biomass is saturated or even reversed beyond a certain threshold. Notably, rhizome length and width were unaffected by all ABA treatments, indicating that ABA primarily influences the number and density of stem-tubers rather than their longitudinal expansion (Figure 2C,D).

### 3.2. Photosynthetic Performance and Chlorophyll Content Are Transiently Modulated by ABA

To better explain the physiological basis of the observed growth responses, leaf chlorophyll content (SPAD value) and gas-exchange parameters were measured over a 9-day period after ABA application (Table 1 and Table 2).

Exogenous ABA induced transient and concentration-dependent changes in photosynthetic performance. The SPAD value generally increased at lower ABA concentrations but decreased at higher concentrations. Specifically, T2 significantly increased the SPAD value on day 3, whereas T3 showed a significant increase on day 6. In contrast, the higher ABA concentration in T4 significantly reduced the SPAD value on day 9, suggesting that prolonged exposure to high ABA levels may inhibit chlorophyll accumulation or accelerate chlorophyll degradation (Table 1 and Table S1).

Gas-exchange parameters showed clear concentration- and time-dependent responses to exogenous ABA (Table 2). On day 1, the response was non-monotonic, rather than a gradual increase with rising ABA concentration. Compared with the control (T1), the lowest ABA treatment (T2, 5 mg/L) slightly increased most parameters, whereas the higher treatments (T4 and T5, 25 and 35 mg/L) reduced Pn, Gs, and Tr, indicating an inhibitory effect of high ABA levels at the initial stage. By day 3, treatment effects became more differentiated, with T5 showing the highest Pn and T4/T5 exhibiting relatively high Ci. On day 6, the moderate ABA treatments (T2–T4) significantly increased Gs and Tr, whereas T5 markedly decreased these parameters, suggesting stronger stomatal limitation under excessive ABA. Changes in Ci further indicated that ABA-induced photosynthetic regulation involved both stomatal and non-stomatal components. Specifically, when Pn and Gs increased simultaneously under moderate ABA levels, improved stomatal conductance likely contributed to enhanced photosynthesis; in contrast, under T5 at day 6, the reduction in Pn despite relatively high Ci suggests the involvement of non-stomatal limitations. By day 9, differences among treatments had largely disappeared, indicating that the effects of exogenous ABA on gas exchange were mainly transient.

### 3.3. Effects of Exogenous ABA on Ginger Quality Components

Carbohydrate and structural components (soluble sugar, starch, crude fiber, and lignin) exhibited a clear concentration-dependent increase (Table 3). The highest ABA concentration (T5) elicited the most dramatic changes, elevating soluble sugar, starch, and lignin contents by 1.23-, 1.61-, and 4.14-fold, respectively, compared to the control (T1). This substantial accumulation of structural carbohydrates suggests enhanced secondary wall thickening under high ABA conditions. In contrast, vitamin C (ascorbic acid) content declined significantly upon ABA treatment, with reductions of 12.6% to 19.4% across all treatment groups compared to the control (T1: 56.15 μg/g), indicating that ABA may accelerate ascorbate turnover or inhibit its biosynthesis.

Notably, the accumulation pattern of 6-gingerol, the primary pungent and bioactive compound in ginger, diverged from other metabolites. Gingerol content exhibited a bell-shaped response curve to increasing ABA concentrations. Moderate treatments, particularly T3, significantly enhanced gingerol accumulation to 849.07 μg/g, representing a 64.2% increase over the control (517.17 μg/g). However, this promotive effect diminished at higher concentrations, with gingerol content in the T5 treatment (553.05 μg/g) not significantly different from that in the control. The rank order of gingerol content across treatments was T3 > T2 > T4 > T5 > T1.

### 3.4. Effects of Exogenous ABA on Endogenous Hormone Content in Ginger

**Figure 3:**
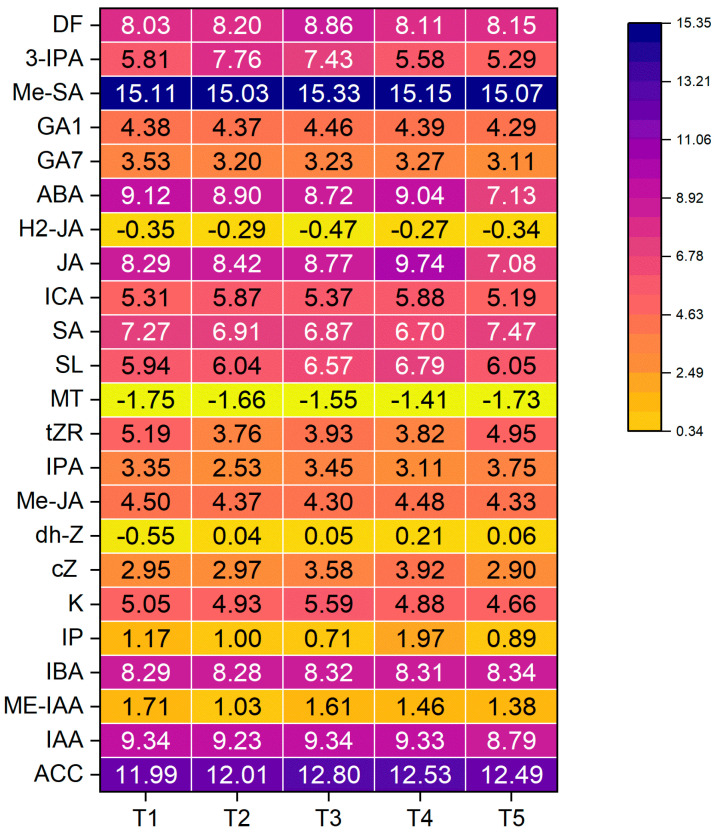
Heatmap of endogenous hormone profiles in ginger rhizomes in response to exogenous ABA. Hormone abundance was quantified by UHPLC-MRM-MS/MS, log2-transformed, and hierarchically clustered. Each row represents a hormone; each column represents a biological replicate. Color scale indicates normalized signal intensity (red: high abundance; blue: low abundance). Hormone abbreviations: ACC, 1-aminocyclopropanecarboxylic acid; IAA, indole-3-acetic acid; ME-IAA, methyl indole-3-acetate; IBA, 3-indolebutyric acid; IP, N6-isopentenyladenine; K, kinetin; cZ, cis-zeatin; dh-Z, dihydrozeatin; Me-JA, methyl jasmonate; IPA, isopentenyl adenosine; tZR, trans-zeatin-riboside; MT, melatonin; SL, strigolactone; SA, salicylic acid; ICA, indole-3-carboxaldehyde; JA, jasmonic acid; H2-JA, dihydrojasmonic acid; ABA, abscisic acid; GA7, gibberellin A7; GA1, gibberellin A1; Me-SA, methyl salicylate; 3-IPA, 3-indole propionic acid; DF, doxifluridine. T1: 0 mg/L ABA (control), T2: 5 mg/L, T3: 15 mg/L, T4: 25 mg/L, T5: 35 mg/L.

To comprehensively evaluate the global reprogramming of endogenous hormone networks in response to exogenous ABA application, hierarchical clustering analysis was performed on the log2-transformed hormone profiles across five treatment groups (T1–T5) (Figure 3), and all hormone content differences were assessed based on one-way ANOVA followed by Duncan’s multiple range test (*p* < 0.05) (Table S2). The heatmap analysis revealed that different ABA treatments altered the accumulation patterns of endogenous hormones and related metabolites in ginger, although the magnitude and direction of the responses varied among compounds. Overall, methyl salicylate (MeSA) showed the highest relative abundance across all treatments, with only minor fluctuations, indicating that its accumulation remained comparatively stable under different ABA concentrations. In contrast, doxifluridine (DF) and 3-indole propionic acid (3-IPA) were more responsive to treatment, with DF reaching a relatively higher level under T3, while 3-IPA accumulated more prominently under T2 and T3 than under the other treatments.

Several classical phytohormones, including GA1, GA7, ABA, JA, SA, SL, IAA, and ACC, also exhibited treatment-dependent variation, suggesting that exogenous ABA application may influence multiple hormone signaling pathways simultaneously. Notably, some metabolites showed relatively low abundance across all treatments, such as melatonin (MT) and H2-JA, implying a weaker response or possible suppression under the tested conditions. Taken together, these results indicate that exogenous ABA induced a substantial reprogramming of the endogenous hormone profile in ginger, with medium ABA concentrations generally eliciting more pronounced changes in several signaling-related metabolites.

### 3.5. ABA-Induced Reprogramming of Secondary Metabolism

**Figure 4:**
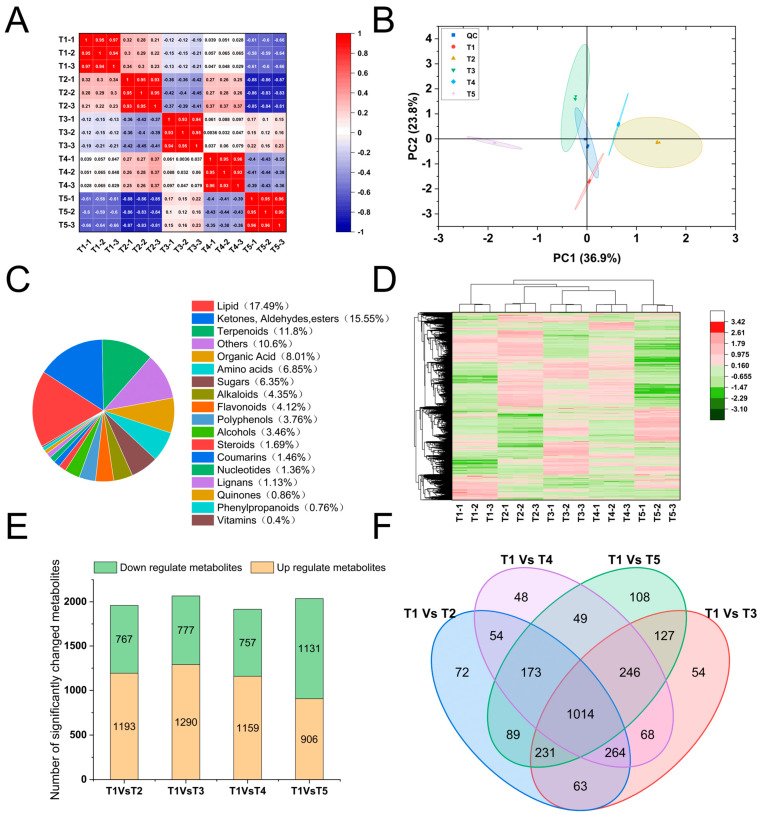
Overview of metabolomic profiling in ginger rhizomes under different ABA treatments. (**A**) Pearson correlation heatmap among all samples. Color intensity represents correlation coefficients (r). Group labels indicate treatment conditions (T1–T5). (**B**) Principal component analysis (PCA) score plot of all samples and quality controls (QCs). PC1 and PC2 explain 34.8% and 21.6% of the total variance, respectively. Each dot represents an individual sample; colors denote treatment groups. (**C**) Pie chart showing the compositional distribution of the 3009 identified metabolites across 18 chemical classes. (**D**) Hierarchical clustering heatmap of all identified metabolites. Each row represents a metabolite; each column represents a sample. Color scale indicates normalized metabolite abundance (red: high; blue: low). (**E**) Bar plot showing the numbers of DEMs in each pairwise comparison. Up-regulated and down-regulated DEMs are shown in red and blue, respectively. (**F**) Venn diagram illustrating the overlap of DEMs among the four comparisons. The central overlapping region (1014) represents metabolites commonly altered by all ABA treatments. T1: 0 mg/L ABA (control), T2: 5 mg/L, T3: 15 mg/L, T4: 25 mg/L, T5: 35 mg/L.

Widely targeted metabolomics identified a total of 3009 metabolites across all samples, classified into 18 major categories, with lipids, terpenoids, and organic acids being the most abundant classes (Figure 4C,D). Principal component analysis (PCA) revealed clear separation among treatment groups, with quality control (QC) samples clustering tightly, confirming the robustness and reproducibility of the dataset (Figure 4A,B).

Differential metabolite analysis uncovered a concentration-dependent metabolic remodeling in response to ABA (Figure 4E). The number of differentially expressed metabolites (DEMs) ranged from 1916 to 2067 across comparisons (T1 vs. T2 to T1 vs. T5), with a core set of 1014 metabolites commonly altered by all ABA treatments (Figure 4F). Notably, the direction of metabolic change shifted with increasing ABA concentration: while low-to-moderate concentrations (T2, T3, T4) predominantly upregulated metabolite accumulation (1159–1290 up vs. 757–777 down), the highest concentration (T5) reversed this trend, resulting in more downregulated (1131) than upregulated (906) metabolites (Figure 4E).

**Figure 5:**
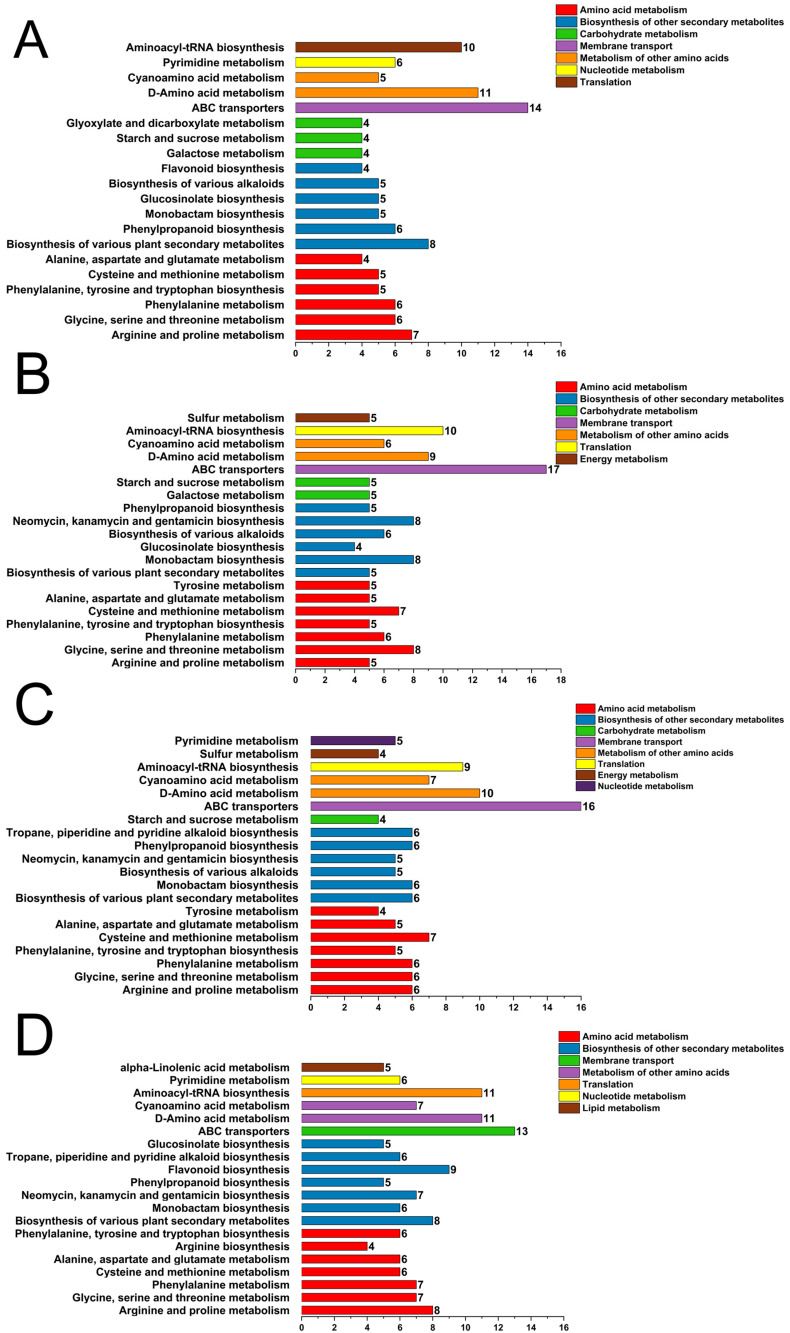
Classification diagram of differential metabolite pathways in T1 vs. T2 (**A**), classification diagram of differential metabolite pathways in T1 vs. T3 (**B**), classification diagram of differential metabolite pathways in T1 vs. T4 (**C**), classification diagram of differential metabolite pathways in T1 vs. T5 (**D**). T1: 0 mg/L ABA (control), T2: 5 mg/L, T3: 15 mg/L, T4: 25 mg/L, T5: 35 mg/L.

KEGG pathway enrichment analysis pinpointed several secondary metabolic pathways that were consistently and significantly modulated by ABA, including the direct pathway responsible for 6-gingerol production, namely stilbenoid, diarylheptanoid, and gingerol biosynthesis, which was significantly enriched across all treatment comparisons with the highest enrichment scores observed in the T3 and T4 groups (Figure 5; Table S3). In addition, upstream pathways that provide precursors for gingerol and other phenolic compounds, specifically phenylpropanoid biosynthesis and flavonoid biosynthesis, were also strongly enriched, suggesting a broad activation of phenylpropanoid metabolism in response to ABA treatment. Furthermore, enrichment of the monoterpenoid biosynthesis pathway indicated that ABA may also influence the volatile terpene profile of ginger, potentially contributing to its aromatic properties. Notably, the plant hormone signal transduction pathway was significantly enriched across comparisons.

### 3.6. Transcriptomic Landscape Underpinning Metabolic Reprogramming

To identify the regulatory genes driving these metabolic changes, we performed RNA-seq analysis on the same samples. High-quality sequencing generated over 112 Gb of clean data, with mapping rates to the *Zingiber officinale* genome exceeding 91% and Q30 scores above 97%, ensuring reliable downstream analysis (Tables S4 and S5). A total of 37,002 unigenes were functionally annotated across multiple databases (Figure S1, Table S6).

**Figure 6:**
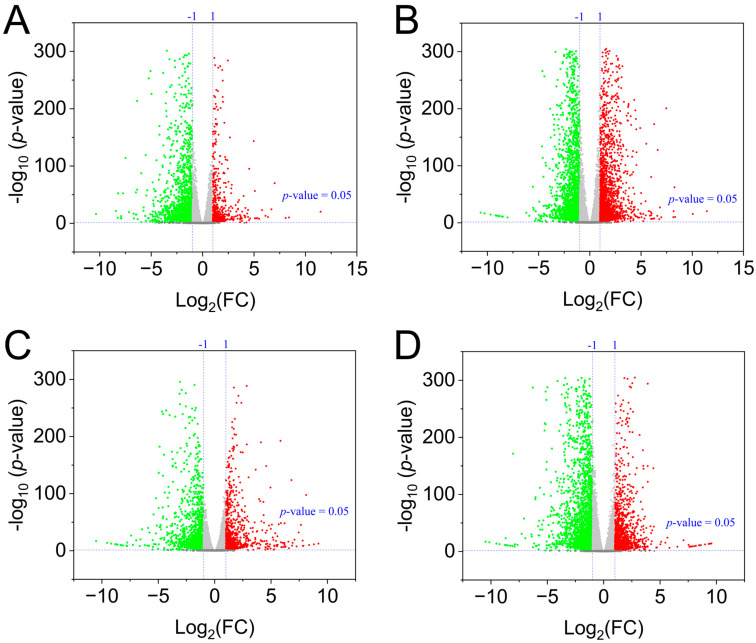
Volcano plot of differential metabolites in T1 vs. T2 (**A**), volcano plot of differential metabolites in T1 vs. T3 (**B**), volcano plot of differential metabolites in T1 vs. T4 (**C**), volcano plot of differential metabolites in T1 vs. T5 (**D**). Green dots represent significantly down-regulated differential metabolites, red dots represent significantly up-regulated differential metabolites, and gray dots represent metabolites with no significant difference. T1: 0 mg/L ABA (control), T2: 5 mg/L, T3: 15 mg/L, T4: 25 mg/L, T5: 35 mg/L.

Differential expression analysis revealed a massive transcriptional reprogramming in response to ABA, with the number of differentially expressed genes (DEGs) ranging from 2175 (T1 vs. T4) to 4773 (T1 vs. T3) (Figure 6, Table S7). Consistent with the metabolome data, the T3 treatment (15 mg/L ABA) elicited the most pronounced transcriptional response, with 2330 up-regulated and 2443 down-regulated genes. The expression of key ABA biosynthetic genes (e.g., *NCED*) (*Maker00028570, Maker00037476*) and cytokinin biosynthesis genes (e.g., *CISZOG*) (*Maker00013266*, *Maker00013351*, *Maker00013354*) were down-regulated (Table S8), underscoring their roles as the optimal concentration for modulating ginger physiology.

**Figure 7:**
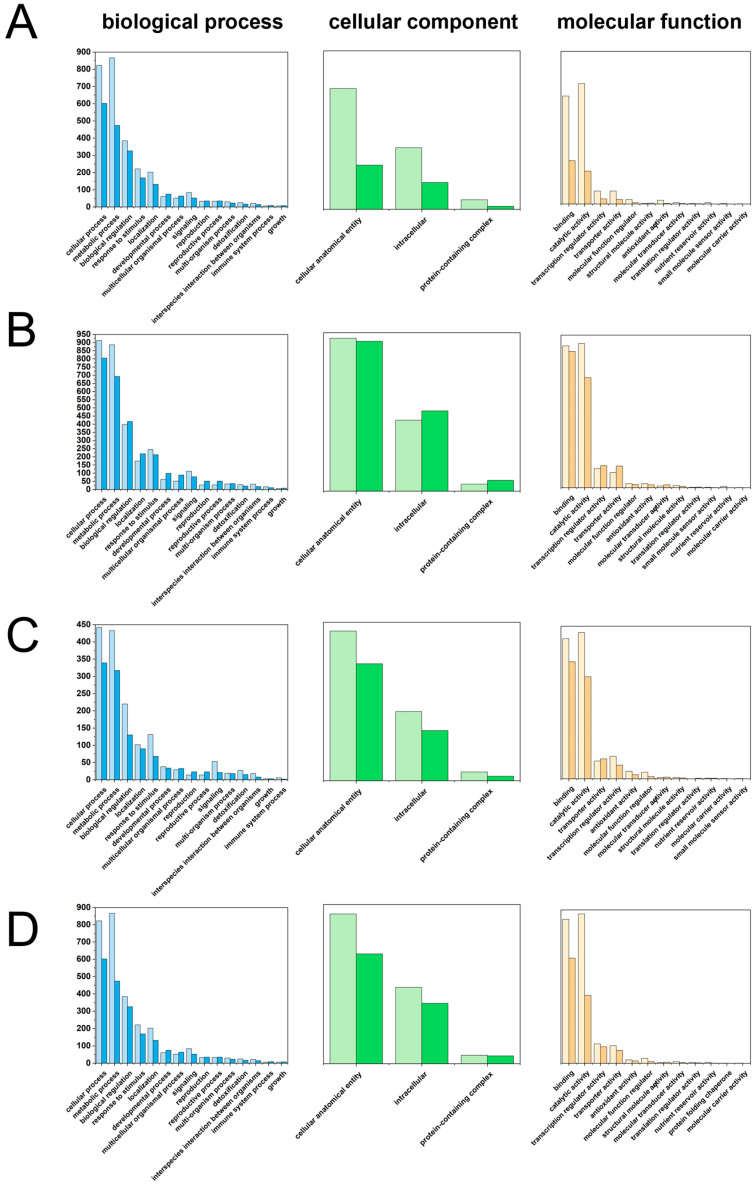
GO enrichment bar chart of DEGs in T1 vs. T2 (**A**), GO enrichment bar chart of DEGs in T1 vs. T3 (**B**), GO enrichment bar chart of DEGs in T1 vs. T4 (**C**), GO enrichment bar chart of DEGs in T1 vs. T5 (**D**). Deep colors (dark blue, dark green, dark yellow) indicate up-regulation, while light colors (light blue, light green, light yellow) indicate down-regulation. T1: 0 mg/L ABA (control), T2: 5 mg/L, T3: 15 mg/L, T4: 25 mg/L, T5: 35 mg/L.

GO enrichment analysis classified DEGs into 35 functional categories, with metabolic process, response to stimulus, and catalytic activity being the most highly enriched terms across all comparisons (Figure 7, Table S9).

**Figure 8:**
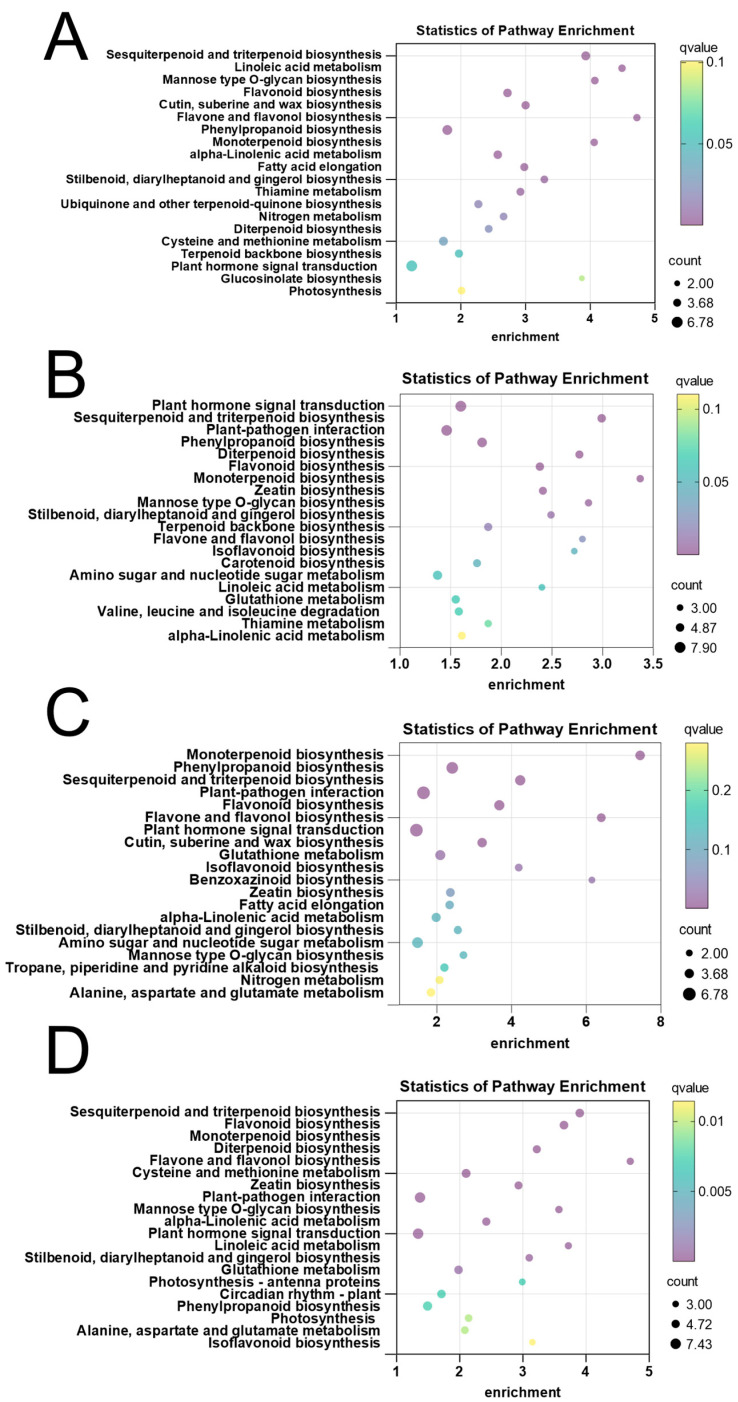
KEGG enrichment bubble plot of DEGs in T1 vs. T2 (**A**), KEGG enrichment bubble plot of DEGs in T1 vs. T3 (**B**), KEGG enrichment bubble plot of DEGs in T1 vs. T4 (**C**), KEGG enrichment bubble plot of DEGs in T1 vs. T5 (**D**). T1: 0 mg/L ABA (control), T2: 5 mg/L, T3: 15 mg/L, T4: 25 mg/L, T5: 35 mg/L.

KEGG pathway analysis of DEGs provided a molecular roadmap for the metabolic changes observed, revealing that the pathways identified as enriched in the metabolome were mirrored at the transcriptional level (Figure 8, Table S10). Plant hormone signal transduction emerged as the most abundantly enriched pathway across all comparisons. Furthermore, the biosynthesis of other secondary metabolites, including phenylpropanoid biosynthesis and stilbenoid, diarylheptanoid, and gingerol biosynthesis, was significantly enriched in all treatment groups, with the most robust enrichment observed in the T3 comparison. In parallel, the metabolism of terpenoids and polyketides emerged as one of the most highly enriched pathways.

### 3.7. Integrated Analysis Links Transcriptional Changes to Metabolic Outcomes

**Figure 9:**
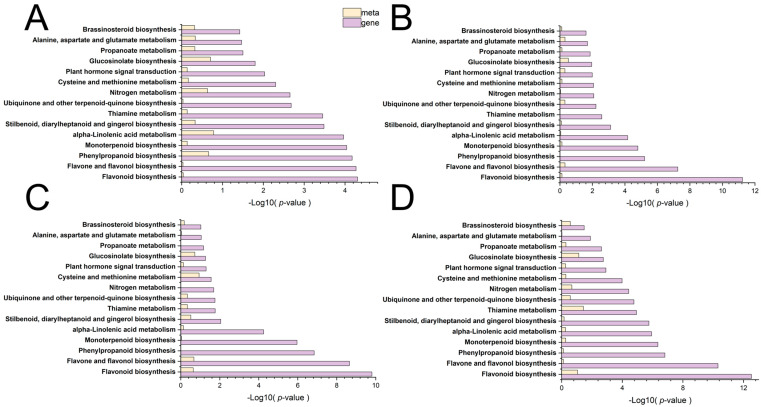
Multi-omics KEGG enrichment analysis reveals convergent pathways modulated by ABA. Bar plots showing the top 30 significantly enriched pathways for DEGs (yellow) and DEMs (purple) in each treatment comparison: (**A**) T1 vs. T2, (**B**) T1 vs. T3, (**C**) T1 vs. T4, (**D**) T1 vs. T5. The *x*-axis represents enrichment significance as −log10(FDR). Pathways highlighted in bold (gingerol biosynthesis, phenylpropanoid biosynthesis, plant hormone signal transduction) are consistently enriched across both omics layers and all treatment groups. T1: 0 mg/L ABA (control), T2: 5 mg/L, T3: 15 mg/L, T4: 25 mg/L, T5: 35 mg/L.

The convergence of metabolomic and transcriptomic data provides compelling evidence for the molecular mechanisms underlying ABA-enhanced ginger quality. Specifically, the coordinated enrichment of gingerol biosynthesis was observed at both the metabolite and gene expression levels across multiple comparisons, with peak enrichment at T3. The strong enrichment of phenylpropanoid biosynthesis in both datasets suggests that ABA not only activates the terminal steps of gingerol production but also enhances the flux through upstream pathways that supply precursor metabolites (e.g., phenylalanine, cinnamic acid derivatives). This systems-level activation of the phenylpropanoid-gingerol biosynthetic network provides a mechanistic explanation for the 64% increase in gingerol content observed in T3-treated plants. Comparative KEGG enrichment analysis of DEGs and DEMs revealed a striking convergence on several key secondary metabolic pathways across all treatment comparisons (Figure 9; Table S11). These convergent pathways included stilbenoid, diarylheptanoid, and gingerol biosynthesis, as well as phenylpropanoid biosynthesis and flavonoid biosynthesis.

### 3.8. A Co-Expressed Gene-Metabolite Network Underlies ABA-Induced Quality Traits

**Figure 10:**
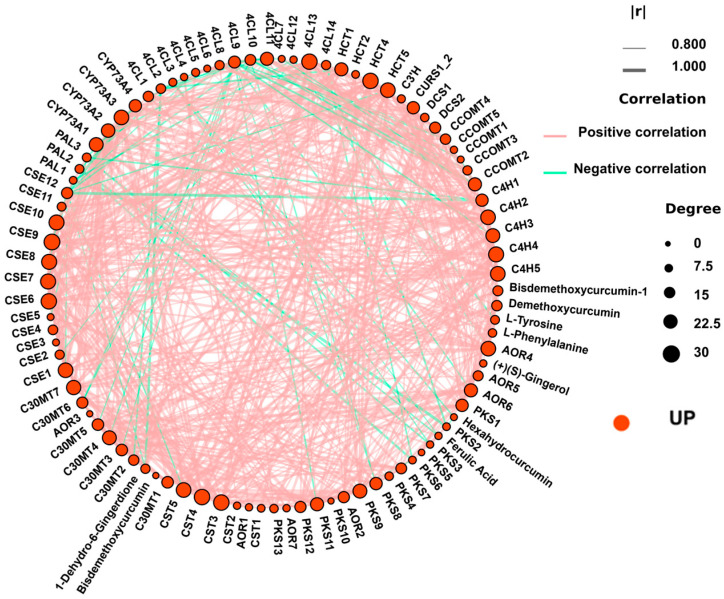
Co-expression network of DEGs and DEMs associated with ABA response. Red dots represent genes and metabolites that are upregulated compared to the control. Edges represent significant positive (red) or negative (green) correlations (|correlation coefficient| > 0.80, *p* < 0.05). The network reveals a highly interconnected hub where hormone signaling genes are tightly linked to secondary metabolic genes and their corresponding metabolites. T1: 0 mg/L ABA (control), T2: 5 mg/L, T3: 15 mg/L, T4: 25 mg/L, T5: 35 mg/L.

The weighted gene co-expression network analysis identified a highly interconnected module containing 583 DEGs that were strongly associated with the accumulation of gingerol-related metabolites (Figure 10; Tables S12 and S13). Within this module, 375 genes were annotated to plant hormone synthesis and signal transduction pathways, including multiple members of the auxin, cytokinin, and jasmonate signaling families. In addition, 121 genes belonged to phenylpropanoid biosynthesis, encoding key enzymes such as *PAL*, *C4H*, and 4-coumarate-CoA ligase (*4CL*), while 45 genes were associated with flavonoid biosynthesis. Furthermore, 21 genes were directly involved in gingerol biosynthesis, including polyketide synthase and oxidoreductase family members, and an additional 21 genes participated in monoterpenoid biosynthesis.

### 3.9. Reconstruction of the Gingerol Biosynthetic Pathway Reveals a Coordinated Upregulation at Optimal ABA Concentration

**Figure 11:**
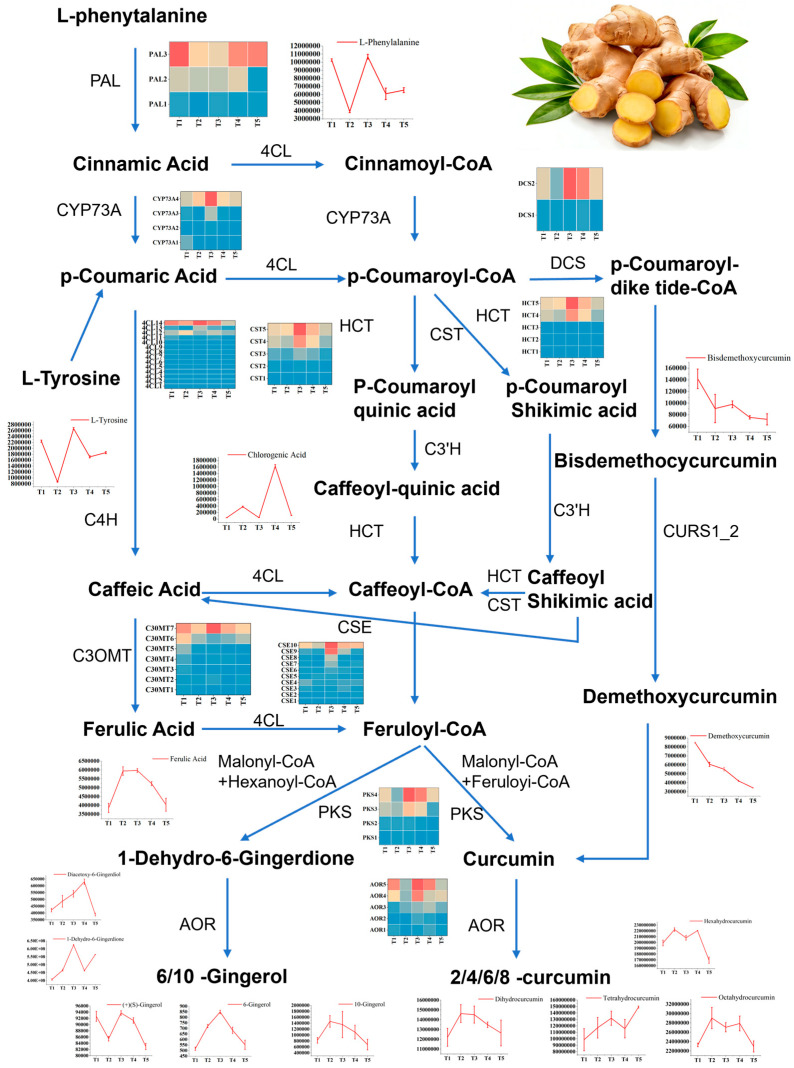
Integrated pathway map of gingerol and curcuminoid biosynthesis under ABA treatment. The schematic illustrates the coordinated transcriptional and metabolic reprogramming in response to optimal ABA concentration (T3). Colored boxes represent differentially expressed genes (DEGs), with red indicating higher accumulation and blue indicating lower accumulation. Metabolite names are shown with line graphs representing log2-fold changes across all five treatments (T1–T5) relative to control. Enzyme abbreviations: PAL, phenylalanine ammonia-lyase; C4H, cinnamate 4-hydroxylase; 4CL, 4-coumarate-CoA ligase; HCT, hydroxycinnamoyl transferase; C3’H, p-coumaroyl shikimate 3’-hydroxylase; CCOAOMT, caffeoyl-CoA O-methyltransferase; CSE, caffeoyl shikimate esterase; C3OMT, caffeic acid 3-O-methyltransferase; PKS, polyketide synthase; AOR, aldo-keto reductase. The pathway demonstrates that T3 treatment optimally activates the entire biosynthetic cascade from phenylalanine to 6-gingerol. T1: 0 mg/L ABA (control), T2: 5 mg/L, T3: 15 mg/L, T4: 25 mg/L, T5: 35 mg/L.

To visualize the flow of genetic information to metabolic output, we mapped all relevant DEGs and DEMs onto the gingerol and curcuminoid biosynthetic pathway, which yielded several critical insights into the concentration-dependent regulation of these bioactive compounds by exogenous ABA (Figure 11). This pathway-level integration revealed that L-phenylalanine and L-tyrosine, the initiating substrates for phenylpropanoid metabolism, accumulated to significantly higher levels under T2 to T4 treatments compared to the control, but declined under the supra-optimal T5 concentration. Furthermore, multiple genes encoding key enzymes in the phenylpropanoid-gingerol pathway exhibited peak expression under the T3 treatment, demonstrating that key biosynthetic genes are transcriptionally co-activated at the optimal ABA concentration of 15 mg/L. Specifically, early pathway genes including *PAL*, *CYP73A*, and *4CL* were significantly upregulated, thereby enhancing metabolic flux from phenylalanine into the phenylpropanoid backbone. In parallel, core modification genes such as hydroxycinnamoyl transferase (*HCT*), p-coumaroyl shikimate 3′-hydroxylase (*C3’H*), and caffeoyl-CoA O-methyltransferase (*CCOAOMT*) showed increased expression, while terminal biosynthesis genes including *PKS* and aldo-keto reductase (*AOR*), which catalyze the final steps of gingerol formation, were maximally induced at T3.

### 3.10. A Working Model for ABA-Mediated Quality Enhancement in Ginger

Integrating all lines of evidence from phenotypic, physiological, hormonal, transcriptomic, and metabolomic analyses, we propose a working model for ABA-mediated growth and quality regulation in ginger, as illustrated in Figure 11. In this model, exogenous ABA application at the optimal concentration of 15 mg/L is perceived and transduced through plant hormone signaling pathways, leading to the transcriptional activation of a co-expressed gene network that coordinately upregulates phenylpropanoid biosynthesis to increase precursor supply and gingerol-specific biosynthesis to convert those precursors into bioactive end products. This concerted regulation results in a 64% increase in 6-gingerol content, enhanced accumulation of related curcuminoids, and improved overall nutritional quality, all achieved without the growth penalties observed at higher ABA concentrations.

## 4. Discussion

### 4.1. ABA Elicits Concentration-Dependent Effects on Ginger Growth by Modulating Source-Sink Dynamics and Hormonal Homeostasis

The pleiotropic effects of ABA on plant growth and development are well-documented, yet its concentration-dependent role in regulating the source-sink relationship in rhizomatous crops remains underexplored. Our study demonstrates that foliar-applied ABA modulates ginger growth in a distinctly non-linear, concentration-dependent manner, with low-to-moderate concentrations (5–15 mg/L) promoting both aboveground vegetative growth (source) and belowground rhizome yield (sink), while high concentrations (≥25 mg/L) diminish these benefits or even prove inhibitory.

The promotion of branching and plant height by 5–15 mg/L ABA (Figure 1) is consistent with reports in rice [33] and pepper [34], where low-dose ABA enhanced tillering and seedling vigor. In contrast, the inhibition of branching at 35 mg/L ABA resembles the responses reported in *Arabidopsis* [35] and tea plants [36], supporting the view that excessive ABA suppresses lateral bud outgrowth. Notably, this inhibitory effect was unlikely to be mediated by SL, because SL levels under T5 were comparable to those in the control and lower than those in T3 and T4 (Figure 3). In addition, exogenous ABA did not cause a proportional increase in endogenous ABA content, even under T5, suggesting that ABA homeostasis may buffer changes in the endogenous ABA pool through feedback regulation of biosynthesis and catabolism. By contrast, the approximately two-fold increase in IP indicates that cytokinin metabolism was more responsive to ABA treatment, which may help sustain shoot development under moderate ABA levels. Meanwhile, the decline in MT suggests a shift in hormonal balance under ABA treatment, possibly reflecting altered stress- and growth-related signaling. Together, these results indicate that the growth response of ginger to exogenous ABA depends not simply on endogenous ABA accumulation itself, but on broader hormone reprogramming involving cytokinin, melatonin, and other signaling pathways. The species-specific insensitivity of leaf length to ABA in our study, in contrast to its promotion in maize [37], further underscores the importance of genetic background and developmental context in determining ABA responsiveness.

Crucially, our results suggest a mechanistic link between aboveground source capacity and belowground sink strength. The optimal T2 (5 mg/L) treatment not only increased branch number (source expansion) but also promoted stem-tuber formation and yield (sink strengthening) (Figure 2), implying improved assimilate partitioning under an appropriate ABA level. This interpretation is consistent with the gas-exchange data (Table 2), which showed that low to moderate ABA treatments transiently promoted photosynthetic performance and stomatal conductance at specific stages, potentially enhancing carbon supply to developing sinks. By day 9, however, most gas-exchange parameters had largely returned to control levels, indicating that exogenous ABA acted mainly as a short-term physiological trigger rather than a sustained regulator, which may help avoid the growth penalties associated with prolonged exposure to high ABA levels [38].

The antagonistic relationship between yield and structural carbohydrate accumulation at high ABA concentrations (T5) is particularly noteworthy. While T5 dramatically increased lignin and starch content (Table 3), indicative of secondary wall thickening and carbon storage, this came at the cost of reduced branch number and gingerol accumulation. This trade-off suggests that under high ABA signaling, carbon flux is redirected from growth and secondary metabolism towards structural reinforcement, a classic stress response that prioritizes survival over productivity [39].

### 4.2. Hormonal Crosstalk Underlies the Concentration-Dependent Actions of Exogenous ABA

A central finding of this study is that exogenous ABA orchestrates a global reprogramming of the endogenous hormone network, providing a mechanistic basis for its pleiotropic effects. The observation that moderate ABA concentrations (T2, T3) elevated levels of growth-promoting hormones—including auxins (IBA, 3-IPA), cytokinins (kinetin, cis-zeatin, IPA), and bioactive gibberellin GA1 (Figure 3)—directly explains the enhanced branching and plant height documented in Section 3.1. This pattern is consistent with findings in adzuki bean, where exogenous ABA increased IAA, GA, and CTK while suppressing endogenous ABA [40]. However, it contrasts with studies in alfalfa where increasing exogenous ABA progressively elevated endogenous ABA and suppressed IAA/GA [41], highlighting that hormonal responses are species-, tissue-, and developmental stage-specific, as noted by Humplík et al. [42].

The concurrent elevation of stress-related hormones (ACC, JA, SA) alongside growth promoters at 15 mg/L ABA (T3) is particularly intriguing. This suggests that optimal ABA primes the plant for both growth and defense, a balanced state that may underpin the concept of “hormonal fitness.” The increase in ACC (ethylene precursor) and JA is also consistent with their known roles in secondary metabolism, providing a plausible link to the enhanced gingerol accumulation observed in T3-treated plants [43].

Perhaps the most striking hormonal finding is the negative feedback regulation of endogenous ABA biosynthesis. Exogenous application did not elevate, and at high concentrations (T5) significantly suppressed, endogenous ABA levels in rhizomes (Figure 3). This feedback inhibition, well-characterized in ABA biosynthesis mutants [44], demonstrates that the plant actively maintains hormonal homeostasis. The 21% reduction in endogenous ABA at T5, coupled with the suppression of IAA and JA, explains the diminished growth and quality at supra-optimal concentrations, revealing a threshold beyond which the regulatory network becomes dysregulated.

### 4.3. Integrated Omics Reveals a Transcriptional-Metabolic Hub Driving Gingerol Accumulation

The multi-omics analysis provides the most compelling evidence for the molecular mechanisms underlying ABA-mediated quality enhancement. The convergence of metabolomic and transcriptomic enrichment on key pathways—phenylpropanoid biosynthesis (ko00940), gingerol biosynthesis (ko00945), and plant hormone signal transduction (ko04075) (Figure 5, Figure 8 and Figure 9)—validates our experimental system and identifies the core regulatory hub.

The concentration-dependent remodeling of the metabolome (Figure 4E), with low-to-moderate concentrations predominantly up-regulating metabolites and the highest concentration reversing this trend, perfectly mirrors the bell-shaped response of gingerol content (Table 3). This metabolic shift aligns with studies in blueberry [25,45] and grape [46], where ABA orchestrated the accumulation of flavonoids and anthocyanins. The specific enrichment of flavonoids (e.g., epicatechins) and phenolic acids (e.g., ferulic acid) under T3 (Figure 11) positions ginger as a model for understanding ABA-regulated phenylpropanoid metabolism, extending findings from tea [47] and pigeon pea [48] to a rhizomatous spice crop.

The co-expression network analysis provided a systems-level view of this regulation (Figure 10), identifying a highly interconnected module containing 375 hormone signaling genes and 166 secondary metabolic genes including *PAL*, *C4H*, *4CL*, *PKS*, and *AOR*, which suggests that ABA activates a transcriptional cascade in which hormone signals such as auxin, cytokinin, and jasmonate likely activate master transcription factors, including bHLH and MYB, that in turn coordinate the expression of entire biosynthetic pathways [49,50]. This hierarchical model is further supported by the up-regulation of *bHLH* genes observed in our dataset, coupled with their well-documented role in activating *C4H* and artemisinin biosynthesis genes [49,51]. Reconstruction of the gingerol biosynthetic pathway offered a more granular view of this cascade, revealing a coordinated multi-tiered transcriptional activation in response to optimal ABA treatment. Specifically, early pathway genes including *PAL*, *C4H*, and *4CL* were coordinately upregulated, thereby increasing metabolic flux from phenylalanine into the phenylpropanoid backbone, while core modification genes such as *HCT*, *C3’H*, and *CCOAOMT* showed increased expression that directs intermediates toward gingerol-specific branches. Concurrently, terminal biosynthesis genes including *PKS* and *AOR* family members, which catalyze the final steps of gingerol formation, were maximally induced, driving the ultimate assembly of 6-gingerol and related compounds. This multi-tiered transcriptional activation provides a mechanistic explanation for the 64% increase in gingerol content observed under T3 treatment, with the concurrent accumulation of upstream precursors, including phenylalanine and ferulic acid, and downstream products, including 6-gingerol, 10-gingerol, and multiple curcuminoids, confirming that the entire pathway is activated rather than individual steps. Such systems-level activation represents a hallmark of coordinated regulatory control and offers a robust mechanistic explanation for our phenotypic observations [52,53,54].

### 4.4. A Working Model and Agricultural Implications

Integrating all lines of evidence, we propose a working model for ABA-mediated growth and quality regulation in ginger (Figure 11). Exogenous ABA, at an optimal concentration (15 mg/L), is perceived and transduced through plant hormone signaling pathways. This triggers a transcriptional cascade involving bHLH and other transcription factors, which coordinately up-regulate the entire phenylpropanoid-gingerol biosynthetic network. The resulting increase in metabolic flux enhances the accumulation of 6-gingerol and related bioactive compounds, improving nutritional and flavor quality. Simultaneously, the modulation of auxin, cytokinin, and gibberellin pathways optimizes source (branching) and sink (rhizome) development, boosting yield without the trade-offs observed at higher concentrations.

At supra-optimal concentrations (≥25 mg/L), negative feedback suppresses endogenous ABA and other growth-promoting hormones, redirecting metabolism towards structural carbohydrate deposition (lignin, starch) at the expense of both growth and secondary metabolite accumulation.

This model has significant agricultural implications. It identifies 15 mg/L as a precision application threshold for optimizing both yield and functional quality in ginger cultivation. The concept of an “ABA optimization window” may extend to other horticultural crops where secondary metabolites determine quality. Furthermore, our findings suggest that exogenous ABA could be used as a tool to study the fundamental mechanisms of source-sink regulation and secondary metabolism, positioning ginger as a valuable model system for spice and medicinal crops.

## 5. Conclusions and Outlook

This study demonstrates that exogenous ABA regulates ginger growth, branching, yield, and gingerol accumulation in a clear concentration-dependent manner. Among the tested treatments, 15 mg/L ABA produced the most favorable overall response, promoting plant growth and branching while increasing rhizome yield and major gingerol components. Integrated physiological, hormonal, transcriptomic, and metabolomic analyses further showed that this response was associated with selective reprogramming of endogenous hormone networks, activation of phenylpropanoid- and gingerol-related pathways, and coordinated changes in key structural genes and transcription factors. In contrast, excessive ABA treatment caused growth inhibition, accompanied by negative feedback on endogenous ABA accumulation and broader disruption of hormone homeostasis. These findings indicate that the beneficial effects of exogenous ABA in ginger depend not on a simple increase in endogenous ABA itself, but on concentration-dependent hormonal and metabolic coordination.

Future work should focus on functional validation of the candidate regulators identified here, particularly transcription factors and biosynthetic genes associated with hormone crosstalk and gingerol accumulation. It will also be important to clarify the spatial and temporal dynamics of ABA-responsive hormone and metabolite changes during rhizome development, and to evaluate the stability and agronomic applicability of the optimal ABA concentration under field conditions. Overall, this study provides a mechanistic framework for understanding ABA-mediated regulation of growth and quality formation in ginger and offers a useful basis for the targeted improvement of medicinal and horticultural traits in this crop.

## Figures and Tables

**Table 1:** Temporal changes in leaf chlorophyll content (SPAD value) in ginger following foliar application of different ABA concentrations.

Treatment Group	Days After Treatment			
	1 d (SPAD)	3 d (SPAD)	6 d (SPAD)	9 d (SPAD)
T1	39.47 ± 1.86 a	37.23 ± 0.94 ab	36.58 ± 1.09 bc	38.43 ± 1.65 ab
T2	35.98 ± 1.86 b	38.25 ± 1.02 a	36.07 ± 1.58 c	36.38 ± 1.30 b
T3	38.87 ± 2.10 a	37.15 ± 1.13 ab	40.60 ± 1.68 a	38.95 ± 1.22 a
T4	37.38 ± 1.71 ab	37.80 ± 2.14 a	37.78 ± 0.52 b	33.12 ± 2.35 c
T5	35.25 ± 1.57 b	36.05 ± 0.64 b	37.10 ± 1.32 bc	38.35 ± 1.83 ab

Note: Data represent mean ± SD from six biological replicates. Different lowercase letters within each time point indicate significant differences among treatments according to Duncan’s multiple range test (*p* < 0.05). T1: 0 mg/L ABA (control), T2: 5 mg/L, T3: 15 mg/L, T4: 25 mg/L, T5: 35 mg/L.

**Table 2:** Dynamics of photosynthetic gas exchange parameters in ginger leaves over 9 days post-ABA treatment.

Parameters/Indicators	Treatment Group	Days After Treatment			
		1 d	3 d	6 d	9 d
Pn (µmol·m^−2^·s^−1^)	T1	4.71 ± 1.62 ab	6.80 ± 0.82 b	6.68 ± 0.20 ab	11.49 ± 1.58 a
	T2	6.84 ± 1.34 a	5.42 ± 0.67 bc	5.02 ± 0.20 bc	10.04 ± 1.52 a
	T3	5.78 ± 1.89 ab	8.42 ± 0.68 a	7.49 ± 1.52 a	11.03 ± 0.64 a
	T4	3.24 ± 0.53 b	5.20 ± 0.87 c	4.23 ± 0.48 c	9.78 ± 1.66 a
	T5	3.23 ± 0.56 b	8.76 ± 0.81 a	5.18 ± 1.53 bc	11.47 ± 1.70 a
Gs (mmol·m^−2^·s^−1^)	T1	22.67 ± 8.32 ab	14.47 ± 1.65 a	26.22 ± 2.60 d	116.73 ± 22.94 a
	T2	33.00 ± 9.68 a	10.24 ± 1.28 b	56.35 ± 3.44 a	111.20 ± 18.40 a
	T3	22.21 ± 5.21 ab	10.93 ± 0.41 b	41.53 ± 1.45 b	110.67 ± 3.51 a
	T4	15.54 ± 2.50 b	6.88 ± 1.09 c	33.49 ± 0.70 c	91.27 ± 26.59 a
	T5	15.96 ± 2.05 b	8.89 ± 1.56 bc	5.28 ± 2.69 e	101.83 ± 23.58 a
Ci (µmol·m^−2^·s^−1^)	T1	103.43 ± 8.37 b	279.09 ± 11.78 d	238.09 ± 25.37 c	598.69 ± 21.28 a
	T2	140.43 ± 30.05 a	388.63 ± 61.95 c	571.53 ± 8.61 a	599.71 ± 28.18 a
	T3	121.71 ± 11.66 ab	334.62 ± 42.84 cd	387.35 ± 11.25 b	601.27 ± 17.00 a
	T4	102.94 ± 4.49 b	493.38 ± 37.93 b	514.14 ± 18.79 a	577.41 ± 30.13 a
	T5	111.62 ± 6.04 ab	647.61 ± 31.65 a	542.60 ± 70.23 a	569.68 ± 13.46 a
Tr (mmol·m^−2^·s^−1^)	T1	1.93 ± 0.65 b	1.21 ± 0.13 a	1.25 ± 0.18 d	4.05 ± 0.54 a
	T2	2.92 ± 0.76 a	0.93 ± 0.11 bc	2.65 ± 0.12 a	4.92 ± 0.75 a
	T3	1.90 ± 0.42 b	0.96 ± 0.05 b	2.13 ± 0.13 b	4.29 ± 0.16 a
	T4	1.39 ± 0.23 b	0.62 ± 0.10 d	1.65 ± 0.03 c	3.83 ± 0.97 a
	T5	1.53 ± 0.19 b	0.74 ± 0.12 cd	0.51 ± 0.27 e	3.83 ± 0.62 a

Data represent mean ± SD from three biological replicates. Different lowercase letters within each time point indicate significant differences among treatments according to Duncan’s multiple range test (*p* < 0.05). Abbreviations: Pn, net photosynthetic rate; Gs, stomatal conductance; Ci, intercellular CO_2_ concentration; Tr, transpiration rate. T1: 0 mg/L ABA (control), T2: 5 mg/L, T3: 15 mg/L, T4: 25 mg/L, T5: 35 mg/L.

**Table 3:** Changes in nutritional quality of ginger under different concentrations of exogenous ABA treatment.

Treatment Group	Crude Fiber (%)	Soluble Sugar (mg/g)	Starch (mg/g)	Lignin (mg/g)	Vitamin C (μg/g)	Soluble Protein (mg/g)	6-Gingerol Content (μg/g)
T1	0.02 ± 0 d	36.48 ± 2.63 d	42.68 ± 0.64 e	41.67 ± 2.08	56.15 ± 1.99 a	16.34 ± 0.54 e	517.17 ± 14.64 c
T2	0.03 ± 0 c	41.09 ± 1.26 d	50.84 ± 0.93 d	56.68 ± 1.17	49.08 ± 2.62 b	19.47 ± 0.78 d	721.53 ± 15.41 b
T3	0.04 ± 0 b	62.77 ± 3.49 c	65.67 ± 2.26 c	80.81 ± 2.22	46.63 ± 1.61 bc	22.78 ± 0.85 c	849.07 ± 14.87 a
T4	0.05 ± 0 a	70.93 ± 5.57 b	77.57 ± 2.70 b	119.85 ± 6.70	45.24 ± 1.00 c	27.84 ± 0.88 b	683.90 ± 28.5 b
T5	0.05 ± 0 a	81.42 ± 3.93 a	111.58 ± 3.92 a	214.52 ± 13.87	45.80± 1.95 bc	31.98 ± 0.46 a	553.05 ± 41.77 c

Data represent mean ± SD from three biological replicates. Different lowercase letters within each time point indicate significant differences among treatments according to Duncan’s multiple range test (*p* < 0.05). Note: T1: 0 mg/L ABA (control), T2: 5 mg/L, T3: 15 mg/L, T4: 25 mg/L, T5: 35 mg/L.

## Data Availability

The original contributions presented in this study are included in the article/Supplementary Materials. Further inquiries can be directed to the corresponding authors.

## Supplementary Materials

The following supporting information can be downloaded at: https://www.mdpi.com/article/10.3390/plants15081228/s1. Figure S1: Functional annotation of all genes and new genes in multiple databases; Table S1: Temporal changes in leaf chlorophyll content (SPAD value) in ginger following foliar application of different ABA concentrations; Table S2: Endogenous hormone contents under different ABA treatments; Table S3: The KEGG pathway enrichment analysis of treatment groups T1–T5; Table S4: Summary of sequencing data quality for each sample; Table S5: Statistics of sequencing reads mapped to the reference genome for each sample; Table S6: Functional annotation statistics of all genes and new genes in multiple databases; Table S7: Statistics of differentially expressed genes (DEGs) between each treatment group and the control (T1); Table S8: Gene expression matrix for all samples; Table S9: GO functional classification statistics of differentially expressed genes in T1–T5 treatment groups; Table S10: KEGG pathway enrichment analysis of differentially expressed genes between each treatment group and the control (T1); Table S11: Comparative KEGG pathway enrichment analysis of differentially expressed genes and differential metabolites between each treatment group and the control (T1); Table S12: List of 583 DEGs in the WGCNA module associated with gingerol-related metabolites; Table S13: Expression matrix of gingerol-related metabolites and key pathway genes across samples.
