# Supplementary figures and images for: Concentration-Dependent Regulation of Ginger Growth and Quality by Abscisic Acid: Insights from Integrated Metabolomic and Transcriptomic Analyses
Source: Plants (Basel). 2026 Apr 16;15(8):1228. doi: 10.3390/plants15081228 (PMC13119435; doi:10.3390/plants15081228)

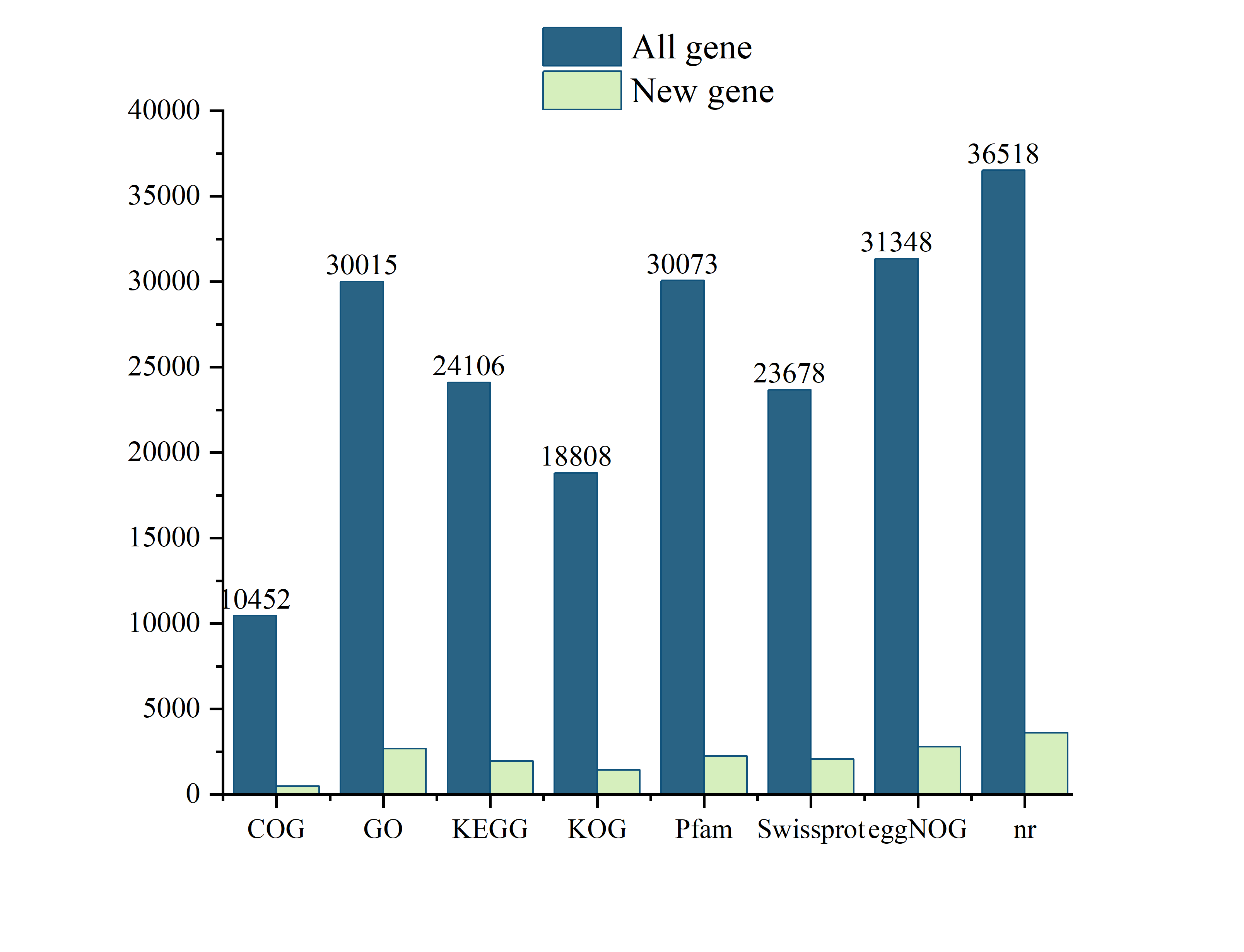

Supplement: Supplementary file 1 [file plants-15-01228-s001.zip › Figure S1.png]
